# Peer-led active tuberculosis case-finding among people living with HIV: lessons from Nepal

**DOI:** 10.2471/BLT.16.179119

**Published:** 2017-02-01

**Authors:** Dipu Joshi, Raisha Sthapit, Miranda Brouwer

**Affiliations:** a Naya Goreto, Kathmandu, Nepal.; b PHTB Consult, Lovensestraat 79, Tilburg, 5014 DN, Netherlands.

## Abstract

**Problem:**

People living with a human immunodeficiency virus (HIV) infection have a high risk of tuberculosis and should undergo regular screening. However, they can be difficult to reach because they are stigmatized and discriminated against.

**Approach:**

In Nepal, the nongovernmental organization Naya Goreto implemented a peer-led tuberculosis screening project in which people living with HIV volunteered to contact others in this high-risk population. Volunteers took part in a short training course, after which they attempted to contact people living with HIV through existing networks and self-help groups. Tuberculosis screening and testing were carried out in accordance with national guidelines.

**Local setting:**

In Nepal, the prevalence of HIV infection is 0.3% in the general population but is much higher, at 6%, in people in Kathmandu who inject drugs. To date, the health system has not been able to implement systematic tuberculosis screening in people living with HIV.

**Relevant changes:**

Between May 2014 and mid-September 2015, 30 volunteers screened 6642 people in 10 districts, 5430 (82%) of whom were living with HIV. Of the 6642, 6046 (91%) were tested for tuberculosis and 287 (4.3%) were diagnosed with the disease, 240 of whom were HIV-positive. Of those with tuberculosis, 270 (94%) initiated treatment.

**Lessons learnt:**

Using peers to contact people living with HIV for tuberculosis screening resulted in a high participation rate and the identification of a considerable number of HIV-positive tuberculosis patients. Follow-up during treatment was difficult in this highly mobile group and needs more attention in future interventions.

## Résumé

**Problème:**

Les personnes qui vivent avec le virus de l'immunodéficience humaine (VIH) courent un risque accru de contracter la tuberculose et devraient se soumettre à un dépistage régulier. Cependant, cela s'avère parfois difficile du fait de leur stigmatisation et de la discrimination à leur encontre.

**Approche:**

Au Népal, l'organisation non gouvernementale Naya Goreto a mis en place un projet de dépistage de la tuberculose par les pairs, qui a consisté à ce que des personnes vivant avec le VIH se portent bénévoles pour contacter d'autres membres de cette population à haut risque. Les bénévoles ont suivi une courte formation, après laquelle ils ont tâché de contacter des personnes vivant avec le VIH par le biais de réseaux existants et de groupes d'entraide. Le dépistage de la tuberculose ainsi que les examens ont eu lieu conformément aux directives nationales.

**Environnement local:**

Au Népal, la prévalence du VIH est de 0,3% dans la population générale mais elle est bien plus élevée (6%) chez les usagers de drogues injectables de Katmandou. À ce jour, le système de santé n'a pas réussi à organiser le dépistage systématique de la tuberculose chez les personnes vivant avec le VIH.

**Changements significatifs:**

De mai 2014 à mi-septembre 2015, 30 bénévoles ont examiné 6642 personnes dans 10 districts, parmi lesquelles 5430 (82%) vivaient avec le VIH. Sur ces 6642 personnes, 6046 (91%) ont été soumises à un test de la tuberculose et la maladie a été diagnostiquée chez 287 (4,3%) d'entre elles, dont 240 séropositives. Parmi les personnes atteintes de tuberculose, 270 (94%) ont commencé un traitement.

**Leçons tirées:**

Le recours à des pairs pour contacter des personnes vivant avec le VIH afin de dépister la tuberculose a permis d'obtenir un fort taux de participation et d'identifier un nombre considérable de patients séropositifs atteints de tuberculose. Le suivi pendant le traitement s'est révélé difficile dans ce groupe extrêmement mobile et nécessitera une attention accrue lors des interventions futures.

## Resumen

**Problema:**

Las personas que viven infectadas con el virus de la inmunodeficiencia humana (VIH) tienen un alto riesgo de sufrir tuberculosis y deberían someterse a exámenes de detección con regularidad. No obstante, pueden resultar difíciles de alcanzar, pues están estigmatizadas y discriminadas.

**Enfoque:**

En Nepal, la organización no gubernamental NayaGoreto implementó un proyecto de exámenes de detección de tuberculosis dirigidos por pares en el cual personas que viven con el VIH se presentaban voluntarias para ponerse en contacto con otras personas en esta población de alto riesgo. Los voluntarios realizaron un pequeño curso de formación, tras el cual intentaron contactar con personas que viven con el VIH a través de redes existentes y grupos de autoayuda. Los exámenes y pruebas de detección de tuberculosis se realizaron según las directrices nacionales.

**Marco regional:**

En Nepal, la prevalencia de la infección por VIH es del 0,3% en la población general, pero es mucho mayor (del 6%) en Katmandú, donde la población consume drogas por vía intravenosa. Hasta la fecha, el sistema sanitario no ha sido capaz de implementar un examen de detección de tuberculosis sistemático en personas que viven con el VIH.

**Cambios importantes:**

Entre mayo de 2014 y mediados de septiembre de 2015, 30 voluntarios hicieron exámenes de detección a 6 642 personas de 10 distritos; 5 430 (82%) de estas personas vivían con el VIH. De las 6 642, 6 046 (91%) se sometieron a pruebas de tuberculosis y 287 (4,3%) fueron diagnosticadas con la enfermedad, 240 de las cuales eran positivas en VIH. De las personas afectadas por tuberculosis, 270 (94%) iniciaron un tratamiento.

**Lecciones aprendidas:**

Al utilizar pares para contactar con personas que viven con el VIH para someterlos a exámenes de detección de tuberculosis, se produjo una alta tasa de participación y se identificó una cantidad considerable de pacientes con tuberculosis y resultados positivos de VIH. El seguimiento durante el tratamiento fue complicado en este grupo altamente cambiante, por lo que es necesaria una mayor atención en futuras intervenciones.

## ملخص

**المشكلة:**

يواجه المصابون بعدوى فيروس نقص المناعة البشرية (HIV) مخاطر عالية للإصابة بالسل، ويجب أن يخضعوا للفحص المنتظم. ومع ذلك، فقد يصعب الوصول إليهم بسبب مواجهتهم للشعور بالخزي والتمييز ضدهم.

**الأسلوب:**

قامت المنظمة غير الحكومية "نايا غوريتو" بتنفيذ مشروع لفحص حالات السل النشط من خلال مقارنة الحالات المناظرة، قام من خلاله المصابون بفيروس نقص المناعة البشرية بالاتصال بالأشخاص المنتمين إلى فئات سكانية تواجه نسب خطر عالية. وشارك المتطوعون في دورة تدريبية قصيرة، يحاولون بعدها الاتصال بالمصابين بفيروس نقص المناعة البشرية من خلال الشبكات القائمة ومجموعات المساعدة الذاتية. وأُجريت فحوصات واختبارات السل وفقًا للمبادئ التوجيهية الوطنية.

**المواقع المحلية:**

بلغ معدل انتشار عدوى فيروس نقص المناعة البشرية في نيبال 0.3‏‏٪ بين عموم السكان ولكن المعدل كان أعلى من ذلك بكثير في كاثماندو حيث بلغ ‏6‏%‏ بين الأشخاص الذين يتعاطون المخدرات بالحقن. حتى الآن، لم يستطع النظام الصحي تنفيذ فحص السل المنهجي للأشخاص الذين يعانون من فيروس نقص المناعة البشرية.

**التغيّرات ذات الصلة:**

قام 30 متطوعًا بفحص 6642 شخصًا في 10 أحياء، كان 5430 (‏‏82‏٪) منهم يعانون من فيروس نقص المناعة البشرية وذلك في الفترة ما بين أيار/مايو 2014 ومنتصف أيلول/سبتمبر 2015. ومن بين 6642 شخصًا، تم توقيع كشف السل على 6046 شخصًا (‏‏91‏‏٪) منهم، وأظهر التشخيص إصابة 287 شخصًا (‏4.3‏‏٪) بهذا المرض، منهم 240 شخصًا يعانون من فيروس نقص المناعة البشرية. وبدأ المصابون بمرض السل الذين يبلغ عددهم 270 (94‏‏‏٪) في تعاطي العلاج.

**الدروس المستفادة:**

أنتج استخدام النظراء للاتصال بالأشخاص الذين يعانون من فيروس نقص المناعة البشرية لفحص مرض السل عن نسبة مشاركة عالية والتعرف على عدد كبير من مرضى السل بفيروس نقص المناعة البشرية. كانت المتابعة أثناء العلاج أمرًا صعبًا وسط هذه المجموعة سريعة التغيير، وبالتالي فإن الأمر يحتاج إلى المزيد من الاهتمام في عمليات التدخل المستقبلية.

## 摘要

**问题:**

人类免疫缺陷病毒 (HIV) 携带者患有结核病的风险很高，应该定期筛检。 但是，由于他们受到侮辱和歧视，所以很难与他们取得联系。

**方法:**

在尼泊尔，非政府组织 Naya Goreto 开展了一项同伴支持结核病筛查项目。艾滋病毒感染者自愿联系该高危人群中的其他人员。 志愿者参加了简单的培训课程，之后他们尝试通过现有关系网络和自助小组联系艾滋病毒携带者。 项目按照国家指南开展了结核病筛查和检测。

**当地状况:**

在尼泊尔，一般人群的艾滋病感染率为 0.3%，但加德满都注射毒品人群的感染率较高，为 6%。 截至目前，医疗系统尚无法在艾滋病毒携带者间开展系统性的结核病筛查。

**相关变化:**

2014 年 5 月至 2015 年 9 月中旬，30 名志愿者筛查了 10 个地区的 6642 人，其中 5430 (82%) 人携带艾滋病毒。 在这 6642 人中，6046 (91%) 人接受了结核病检查，287 (4.3%) 人诊断患有此疾病，其中 240 人为 HIV 阳性。 在患有结核病的人中，270 (94%) 人开始了治疗。

**经验教训:**

通过同伴联系艾滋病毒携带者开展结核病筛检项目的参与率很高，并且确定了相当多的 HIV 阳性结核病患者。 在这个具有高度流动性的群体间开展治疗跟进工作很有难度，并且需要在未来的干预中给予更多关注。

## Резюме

**Проблема:**

Люди, живущие с вирусом иммунодефицита человека (ВИЧ), подвергаются высокому риску заражения туберкулезом и должны регулярно проходить скрининговое обследование. Однако может быть сложно установить контакт с такими людьми, поскольку они являются объектами стигматизации и дискриминации.

**Подход:**

В Непале неправительственная организация Naya Goreto реализовала проект скринингового обследования на туберкулез под руководством лиц одного круга с обследуемыми, в рамках которого люди с ВИЧ на добровольной основе устанавливали контакт с другими членами этой группы населения высокого риска. Добровольцы прошли небольшой курс подготовки и затем попытались установить контакт с людьми, живущими с ВИЧ, посредством существующих социальных сетей и групп взаимопомощи. Скрининговое обследование и тестирование на туберкулез проводились в соответствии с национальными руководящими принципами.

**Местные условия:**

В Непале распространенность ВИЧ-инфекции составляет 0,3% от общей численности населения, но гораздо выше (6%) среди жителей Катманду, употребляющих инъекционные наркотики. До сих пор в рамках системы здравоохранения не удавалось обеспечить выполнение систематического скринингового обследования на туберкулез среди людей, живущих с ВИЧ.

**Осуществленные перемены:**

В период с мая 2014 года до середины сентября 2015 года 30 добровольцев провели скрининговое обследование 6642 людей в 10 районах, 5430 (82%) из них жили с ВИЧ. Из 6642 людей 6046 (91%) прошли тестирование на туберкулез, и у 287 (4,3%) он был диагностирован, у 240 из них был позитивный результат анализа на ВИЧ. 270 людей (94%) из числа тех, у кого диагностировали туберкулез, начали лечение.

**Выводы:**

Привлечение лиц одного круга для установления контактов с людьми, инфицированными ВИЧ, в целях проведения скринингового обследования на туберкулез позволило добиться высокого уровня участия и идентифицировать значительное число людей с позитивным результатом анализа на ВИЧ, больных туберкулезом. Сложность проведения последующего врачебного наблюдения во время лечения в этой группе людей была обусловлена ее высокой мобильностью, и в будущих вмешательствах последующему наблюдению необходимо уделять больше внимания.

## Introduction

The prevalence of tuberculosis is elevated in people living with a human immunodeficiency virus (HIV) infection and, in 2014, almost 400 000 HIV-infected people died from tuberculosis globally.^1^ Consequently, the World Health Organization (WHO) recommends systematic tuberculosis screening for people living with HIV.^2^ In Nepal, the National Tuberculosis Center struggles to screen these people even though it adapted its strategies on tuberculosis and HIV infection in 2009. A better way of reaching people living with HIV for tuberculosis screening and treatment would reduce the burden of disease and death. The Stop TB Partnership recommends that people from key populations affected by tuberculosis should be involved in tuberculosis care.^3^

In Nepal, a nongovernmental organization established by and working with people living with HIV and people who use drugs – Naya Goreto – recruited volunteers among people living with HIV for tuberculosis screening of their peers. The project targeted 10 districts with large numbers of people living with HIV. The aims were to screen around 7050 people living with HIV and to ensure that those diagnosed with tuberculosis started treatment. In addition, it was hoped that the project would increase awareness of the importance of tuberculosis screening in people living with HIV among both those affected and health-care workers. In 2013, the 10 project districts accounted for 10 472 tuberculosis patients – 31% of the country’s total.

## Local setting

In Nepal, HIV infection is a relatively small problem: the estimated prevalence in the adult population is 0.3%.^4^ However, the prevalence is much higher in certain groups, such as drug users, migrant workers and sex workers. In Kathmandu, the prevalence in injection-drug users is 6%.^5^ Among female and male sex workers, it is 2% and 9%, respectively.^5^ Many people living with HIV or in these high-risk groups are marginalized, stigmatized and experience discrimination.^6^^–^^8^ For 2013, WHO estimated that the number of incident, HIV-positive tuberculosis patients in Nepal was 1587.^9^ However, of the 33 834 new patients actually reported in the country, only 11% knew their HIV-infection status and only 65 tuberculosis patients were known to be HIV positive, which indicates that tuberculosis is often missed in people living with HIV.

## Approach

In 2014, staff at Naya Goreto asked leaders of self-help groups for people living with HIV and drug users to act as volunteers for the tuberculosis screening project because they knew how to reach people living with HIV. Naya Goreto staff themselves are people living with HIV and former drug-users and are members of these networks and self-help groups. In addition, the organization has worked with some of these leaders in the past. For the intervention, Naya Goreto provided training for volunteers on tuberculosis screening and on how to reach out to HIV-infected people. A one-day training course was given in each district on: (i) the appropriate circumstances for disclosing HIV-infection status; (ii) behavioural change techniques for use with self-help groups and individuals; (iii) maintaining confidentiality; (iv) ensuring the availability of, and access to, diagnostic services without fear of discrimination; and (v) referral for tuberculosis treatment and follow-up.

In collaboration with the National Tuberculosis Center, Naya Goreto developed a screening tool for tuberculosis. The criteria were: (i) a cough for 2 weeks; (ii) fever; (iii) loss of appetite; (iv) weight loss; and (v) no tuberculosis test within the last six months. People living with HIV were contacted by volunteers through existing networks and self-help groups. The volunteers applied the screening tool and encouraged people who met one or more of the five criteria to request further tuberculosis screening at HIV treatment centres or care homes for HIV-infected people. If necessary, Naya Goreto provided money for transport. For the few people who did not want to go to health facilities because of previous poor experiences with formal health-care services, volunteers carried out screening and collected sputum samples using specially provided containers at a place chosen by the individual being screened.

Tuberculosis tests were carried out in accordance with national guidelines: two sputum samples were examined by smear microscopy and a third underwent the rapid molecular diagnostic test GeneXpert^®^ MTB/RIF (Cepheid Inc., Sunnyvale, United States of America). If necessary and available, a chest X-ray or fine-needle aspiration was carried out. Physicians diagnosed some tuberculosis patients clinically. Naya Goreto paid the diagnostic costs, which included a nominal fee for the hospital visit that all patients must pay, and fees for additional examinations. If a person was diagnosed with tuberculosis, a volunteer informed the person and arranged for him or her to attend the tuberculosis treatment facility of their choice. Thereafter, the volunteer tried to remain in contact with the patient and collect information on treatment outcomes.

Naya Goreto collected data on screening, the diagnostic tests carried out, tuberculosis diagnoses, the treatment given and the risk group of each individual who underwent tuberculosis testing. Project coordinators in each district collected information from volunteers and reported to the project manager in Kathmandu, who maintained a database on the test results, treatment and outcomes for all patients with tuberculosis. Participation in screening was voluntary and Naya Goreto obtained informed consent from all participants for use of their data.

## Relevant changes

The tuberculosis screening project was implemented between May 2014 and mid-September 2015. The 30 volunteers (21 male) screened 6642 people, of whom 5430 (82%) were living with HIV. In total, 6046 (91%) of the 6642 were tested for tuberculosis: 5402 were living with HIV, 331 were drug users, 170 were family members of tuberculosis patients, 138 were migrant workers and 5 were slum dwellers. Table 1 shows the tests performed and the results obtained. Overall, 287 tuberculosis patients (205 male and 240 HIV positive) were identified, 270 (94%) of whom started treatment. By the end of the project, the outcome of tuberculosis treatment was known for 178 patients: 39 (22%) successfully completed treatment, 15 (8%) died and 124 (70%) had transferred out of the intervention districts. The remaining 92 patients were still on treatment. The total cost of the project was 132 596 United States dollars, which comprised 61% for diagnostic and transport costs, 20% for Naya Goreto’s expenditure and 12% for training volunteers.

**Table 1:** Tuberculosis tests used to screen people living with HIV, Nepal, 2014–2015

Test	No. tested	No. who tested positive for tuberculosis
Sputum smear test only	3932	106
GeneXpert^®^ MTB/RIF test only	1731	62
Chest X-ray only	42	8
Sputum smear and GeneXpert^®^ MTB/RIF tests	236	49
Sputum smear test and chest X-ray	18	4
Chest X-ray and GeneXpert^®^ MTB/RIF test	20	0
Sputum smear and GeneXpert^®^ MTB/RIF tests and chest X-ray	2	2
Fine-needle aspiration only	17	13
Fine-needle aspiration and chest X-ray	1	1
Clinical diagnosis, including extrapulmonary tuberculosis	47	42
**Total**	**6046**	**287**

HIV: human immunodeficiency virus.

## Lessons learnt

Box 1 summarizes the main lessons learnt from this project. The peer-led, active, tuberculosis case-finding intervention had several successes. First, within a short period the volunteers contacted many people living with HIV, a marginalized group subject to discrimination in Nepal. Second, many more HIV positive tuberculosis patients were identified than notified at a national level in 2013.^9^ Third, no major problem was encountered with using peer volunteers to reach this key population. Fourth, reports from volunteers indicated that some screening was still taking place after the project ended.

Box 1 **Summary of main lessons learnt**

• Using peer volunteers to contact people living with human immunodeficiency virus (HIV) for tuberculosis screening resulted in a high participation rate and the identification of tuberculosis patients.

• Detailed information on tuberculosis screening, the diagnostic tests carried out, the tuberculosis patients identified and their treatment could be obtained by training and monitoring peer volunteers.

• Follow-up during tuberculosis treatment may be difficult because people living with HIV move frequently.

Many people living with HIV in Nepal are former or current drug users and know each other through drug users’ networks. This familiarity contributed to the success of the intervention because the target group trusted the volunteers, whereas they do not always trust health-care workers because of stigmatization and discrimination. Although our project focused on people living with HIV, because volunteers contacted these individuals through their networks and self-help groups, they also reached other groups with a high risk of tuberculosis, such as drug users and migrant workers. We were unable to assess the effectiveness of peer-led screening in these groups. Working with peers has also proved successful in other programmes involving individuals at a high risk of tuberculosis or HIV infection. In the Democratic Republic of the Congo, a peer-led strategy led to increased tuberculosis case-finding among internally displaced and migrant people.^10^ Peer educators chosen from among presumptive tuberculosis patients in these communities identified other community members with presumptive tuberculosis for referral to screening and testing services in health centres.

One limitation of the intervention is that it was externally funded. Most of the project’s expenditure was for diagnostic costs and transport support for patients, who may not have been able to cover these costs themselves. This may have contributed to the project’s success. Although costs could be reduced by integrating tuberculosis screening into routine care for people living with HIV, they may still present a barrier to the successful diagnosis and treatment of tuberculosis. Additional support, such as cash transfers to patients, may help.^11^

A worrying observation was that 70% of people whose tuberculosis treatment outcomes could be assessed at the end of the project had moved out of the intervention districts. Further follow-up of these patients was not possible within the project and they may not all have continued treatment at a health-care facility. Many people living with HIV in Nepal move frequently from place to place because they face discrimination or are migrant workers.^6^ Efforts should be made in future interventions to ensure that these people finish tuberculosis treatment. Contacting peer volunteers at their destination to ensure follow-up may be one solution.
